# Associations of weight-adjusted-waist index and systemic immune-inflammatory index with sKlotho: evidence from the NHANES 2007-2016

**DOI:** 10.3389/fendo.2025.1544796

**Published:** 2025-05-01

**Authors:** Mingming Yang, Qiong Xu, Linjuan Huang, Luqian Zhu

**Affiliations:** Hangzhou TCM Hospital Affiliated to Zhejiang Chinese Medical University, Hangzhou, China

**Keywords:** sKlotho, WWI, SII, aging, mediating effect, NHANES

## Abstract

**Background:**

Klotho is an anti-aging protein, and obesity and inflammation have been identified as having a relationship with Klotho. This study investigated the correlation between weight-adjusted-waist index(WWI), systemic inflammation index(SII) and soluble Klotho (sKlotho) and explored the potential mediating role of SII.

**Methods:**

The association between WWI, SII, and sKlotho was investigated using weighted multivariate linear regression, subgroup analysis, and smoothed curve fitting in this cross-sectional study, which was based on NHANES data. The Bootstrap method was used to test for mediation effects. Covariate selection was validated using the variance inflation factor (VIF).

**Results:**

The study involved a total of 9,506 participants. The results showed that WWI (β=-0.03; 95%CI:-0.04,-0.01; *p*<0.0001) was associated with SII (β=-0.00, 95%CI:-0.00,-0.00:*p*<0.001) and Ln-sKlotho.There was a negative correlation between both WWI and SII and sKlotho. Subgroup analysis showed that gender, age, education, marital status, family income, HEI, PA, hypertension, diabetes, CKD, alcohol use, smoking, and SII did not affect the negative correlation between WWI and sKlotho (FDR-corrected *p*>0.1). The mediation analysis showed that SII played a significant mediating effect in the role of WWI on sKlotho, with SII mediating 6.78% of the relationship between WWI and sKlotho.

**Conclusion:**

There is a negative correlation between WWI and sKlotho, and SII may be an important mediator between WWI and sKlotho. Reducing inflammatory conditions in obese populations may increase sKlotho levels, which in turn may delay organismal aging in middle-aged and elderly people.

## 1 Introduction

With the increasing trend of population aging, the growing number of elderly people and the increasing number of age-related diseases, it is expected that the healthcare burden of age-related diseases will similarly increase (1). The Klotho gene was first noted in 1997 by Kuro-o et al. Defective expression of the Klotho gene in mice leads to syndromes similar to those seen in human aging, including a short lifespan, infertility, Atherosclerosis, skin atrophy, osteoporosis, and emphysema (2). Xu Y et al. showed that the Klotho gene is responsible for encoding a transmembrane protein (α-Klotho) (3). The extracellular structural domain of α-Klotho can be shed to form a soluble variant of the gene called soluble Klotho (sKlotho), which is widely found in urine, blood, and cerebrospinal fluid (CSF) (3–7). sKlotho possesses inflammation-regulating, antioxidant, and senescence-preventive functions, which make it a promising target for therapeutic development in aging-related diseases (8–11). Therefore, the study of the factors affecting the level of sKlotho will be of great significance in delaying aging and preventing aging-related diseases.

A growing number of studies have shown a relationship between obesity and aging. Daniela Frasca investigated the region of overlap between obesity and aging, suggesting that obesity may be a biomarker of accelerated aging in human B-cell function and antibody response (12). Valentina Salvestrini in her study suggested that obesity may accelerate the rate of aging affecting all aspects of physiology and thus shorten life expectancy and healthy lifespan (13). Santos AL et al. in their study illustrated the description of obesity as similar to the metabolic dysregulation observed in normal aging, suggesting that obesity may accelerate the aging process (14). The National Health and Nutrition Examination Survey (NHANES) database shows that weight gain over 10 years period promotes aging in adulthood (15). Charalampos Lampropoulos et al. showed that obesity is an inflammatory state, characterized with dysregulation of gastrointestinal hormones, such as GLP-1, ghrelin and PYY, leading also to impaired glucose homeostasis (16), all of which negatively impacts life expectancy. Abdominal obesity, which reflects the amount of visceral fat, is one of the important phenotypes of unhealthy metabolism (17–19). Previously, BMI and WC were commonly used as measures of obesity, but in recent years, studies have pointed out that BMI is unable to differentiate between lean body mass and fat mass, and WC does not take into account differences in height and is therefore insufficient in predicting obesity (20–23). Therefore, in 2018 researchers introduced a new obesity index called the weight-adjusted-waist index (WWI), which takes body weight into account and reflects weight-independent central obesity (24). A growing number of studies have shown that WWI has a higher accuracy in predicting metabolic diseases compared to other obesity indices, and Xiaowan Li et al. found that WWI was the best obesity index for predicting CKD and albuminuria when compared to other obesity indices (BMI, WHTR, WC, height, and body weight (25). To the best of our knowledge, no studies of WWI and sKlotho levels have been formally published in the literature.

Inflammation is an influential factor in sKlotho levels. Typiak MA-O et al. showed that lower sKlotho levels are associated with an increased risk of chronic inflammation (26). In addition, Martín-González CA-O et al. illustrated that higher Klotho levels were associated with improved immune system function and reduced oxidative stress, both of which play a key role in the development of inflammation (27). It is well known that there is a complex association between inflammation and obesity. On the one hand, obesity triggers a state of chronic low-grade inflammation, and the abnormal accumulation of macrophages (known as adipose tissue-associated macrophages or ATMs) in adipose depots and other immune cells is an important factor in obesity-induced inflammation (28, 29) On the other hand, chronic low-grade inflammation affects metabolism and may lead to an increase in food intake, thus exacerbating obesity (30, 31). The Systemic Immune Inflammation Index (SII) is considered a new and reliable index that can be used to comprehensively measure systemic immune and inflammation levels in subjects (32–34). Given the relationship between WWI, SII, and sKlotho, SII may play a role in the association between WWI and sKlotho.

Therefore, this study investigated the relationship between WWI, SII, and sKlotho in people over 45 years of age in the United States. To our knowledge, this is the first study to assess the relationship between WWI, SII, and sKlotho. In addition, this study hypothesized that SII plays a mediating role in the relationship between WWI and sKlotho. Based on exploring the relationship between WWI, SII and sKlotho, the role of SII in the relationship between WWI and sKlotho was analyzed.

## 2 Materials and methods

### 2.1 Survey description

The National Center for Health Statistics (NCHS) performed a countrywide research (NHANES) to evaluate the state of nutrition and health in the United States, which served as the basis for the cross-sectional data. Data on demographics, diet, examinations, lab work, and questionnaires were submitted by the participants. All full NHANES research designs and data are available to the public at www.cdc.gov/nchs/NHANEs/. The cross-sectional study complied with the Strengthening the Reporting of Observational Studies in Epidemiology (STROBE) reporting requirements.

### 2.2 Study population

**Figure 1:**
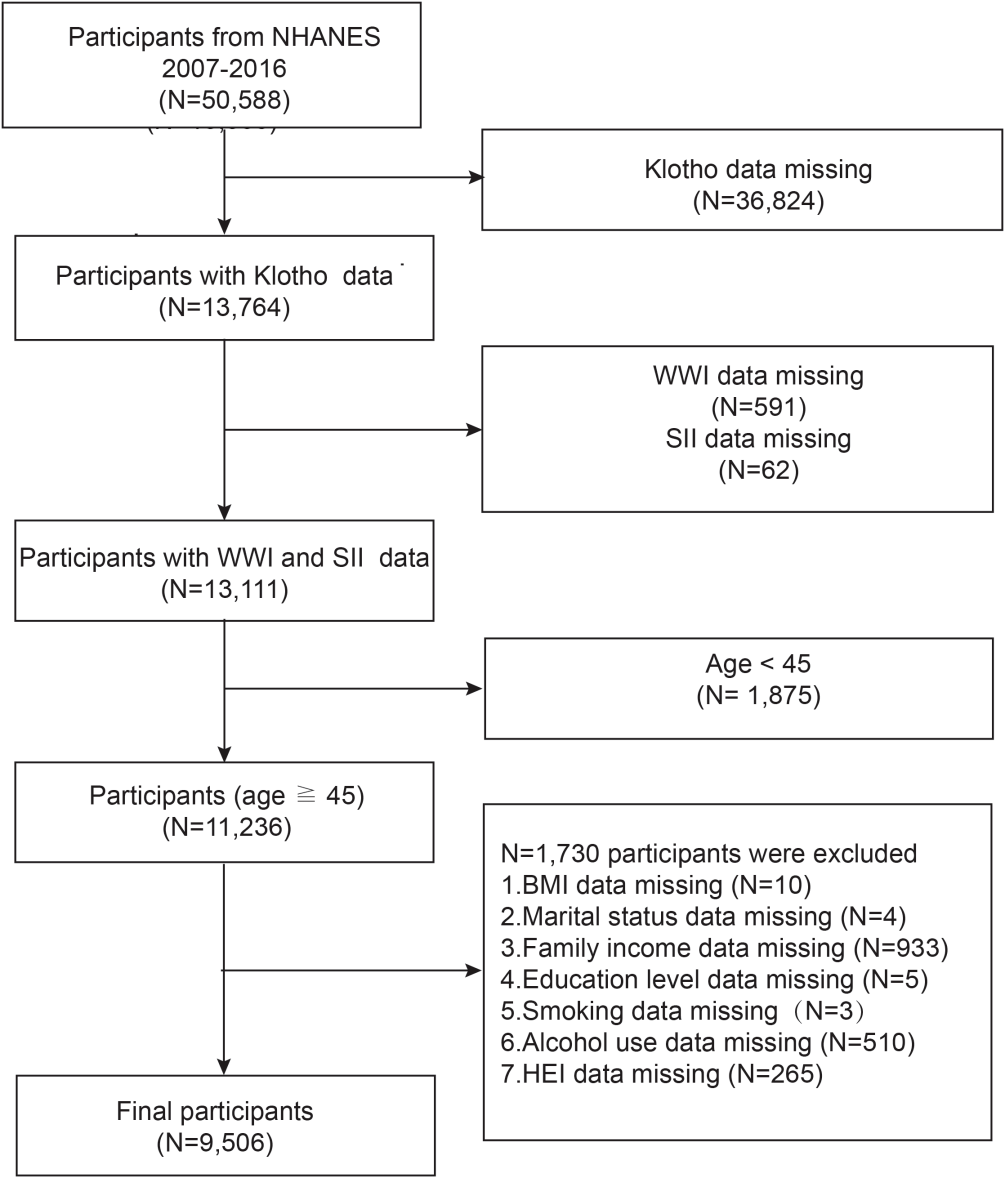
Flowchart of study population inclusion in 2007–2016 NHANES. NHANES, National Health and Nutrition Examination Survey; WWl, weight-adjusted-waist index; SII, systemic immune-inflammation index.

After a comprehensive search and screening of the NHANES database, participants from 2007 through 2016 were included in this study (sKlotho data were not available for other years). Since the focus of this paper is to examine the relationship between WWI, SII, and sKlotho in a population of middle-aged and elderly Americans, exclusion criteria for this study included (a) participants with incomplete information on sKlotho(n = 36,824), (b) participants with incomplete information on WWI (n = 591) or SII(n = 62), (c) participants aged <45 years (n = 1,875), (d) participants with missing data on body mass index (BMI), education, marital status, family income, smoking, alcohol use, and Healthy Eating Index (HEI) (n= 1,730). A total of 9,506 individuals qualified for analysis, as shown in **Figure 1**.

### 2.3 Measurement of sKlotho levels

Blood samples were collected from participants aged 40 to 79 years, transferred, and stored at -80°C during five cycles of the NHANES program. Serum Klotho concentrations were measured using commercially available ELISA kits from a CDC-accredited laboratory (IBL International, Japan) (35). All serum samples were analyzed twice for Klotho concentration, and the average of these two measured concentrations was used as the final measurement. Sample information, experimental measurement details, and quality control for Klotho concentration measurements can be found on the official NHANES Website.

### 2.4 Assessment of systemic immune-inflammation index

SII is an automated hematology analysis tool (CoulterDxH 800 analyzer) that is used to measure platelet count, neutrophil count, and lymphocyte count as a composite index. It is utilized as an exposure variable, and computed by multiplying by the neutrophil count after dividing the lymphocyte count by the platelet counts (36–39).

### 2.5 Assessment of covariates of interest

Demographic covariates in our study included gender, age, race, education level, marital status, and family income. Body measure covariates included BMI. Dietary covariates included energy and the Healthy Eating Index-2015(HEI-2015), HEI-2015 scores range from 0-100, with higher HEI scores reflecting better diet quality. We utilized Day 1 Total Nutrient Intake (DR1TOT) to calculate the 13 components of HEI-2015 and HEI-2015 scores of less than 50, between 50 and 70, and greater than 70 were categorized as insufficient, average, and optimal (40). Associated diseases included chronic kidney disease (CKD), diabetes, and hypertension. Participants with an eGFR ≤ 60 ml/min/1.73m^2^ or a urinary albumin/creatinine ratio ≥ 30 mg/g were defined as having CKD, where the eGFR was calculated by using the formula developed by the Chronic Kidney Disease-Epidemiology Collaboration (CKD-EPI) (41). Diabetes includes self-reported diabetes, use of antidiabetic medications or insulin, glycosylated hemoglobin (HbA1c) level ≥ 6.5%, and fasting blood glucose level ≥ 126 mg/dL. The 2017 American Heart Association/American College of Cardiology (AHA/ACC) guidelines recommend that individuals with SBP ≥130 mmHg and/or DBP ≥80 mmHg be defined as hypertensive (42). Participants were considered to have high blood pressure if they answered yes to any of the following questions: “Have you ever been told by a doctor or other health professional that you have hypertension, also called high blood pressure?”; “are you now taking prescribed medicine for high blood pressure?”; or if they had a high biological measurement value (systolic blood pressure ≥ 130 mm Hg and/or diastolic blood pressure ≥ 80 mm Hg). Questionnaire variables also included alcohol use, smoking, and physical activity (PA). NHANES participants self-reported their physical activity (PA) information using the Global Physical Activity Questionnaire (GPAQ), a validated PA monitoring tool (43). Participants who did not have any PA and were <600 MET per week were categorized as inactive (44), those participants with ≥600 METs per week were categorized as active. Individuals can access all comprehensive NHANES research designs and data at www.cdc.gov/nchs/NHANEs/.

### 2.6 Assessment of weight-adjusted waist circumference index

A sample of NHANES participants’ WWI was obtained from the examination data. The participant’s waist circumference was divided by their weight’s square root to calculate their WWI (45). Following computation using this formula, the study population’s WWI range was 8.59–14.79, with kilograms as the weight unit and centimeters as the waist circumference measurement unit.WWI was considered a continuous variable and was designed as an exposure variable in our study.

### 2.7 Statistical analysis

**Figure 2:**
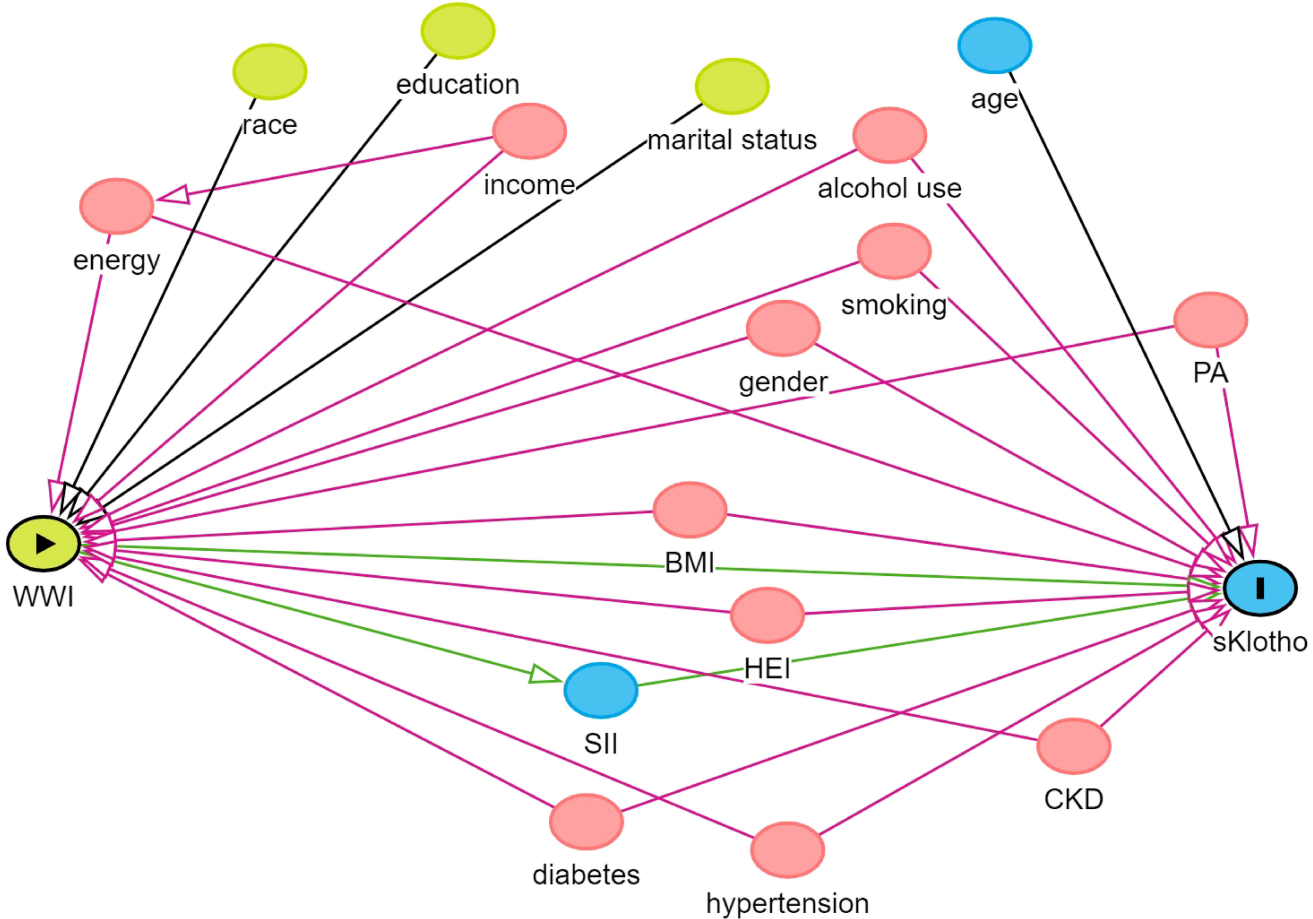
Directed acyclic graphs for the causal effect of WWI on sKlotho.

In descriptive analysis, the compound weighting method is the basis of data description and statistical analysis. Categorical characteristics are expressed as percentages, while continuous variables are expressed as means and standard errors (SE). We constructed three models for weighted multivariate linear regression analysis for different confounders to assess the relationship between sKlotho, WWI and SII in various models. Of these, Model 1: did not adjust for any confounders; Model 2: adjusted for age, gender, and ethnicity; and Model 3: addressed the issue of multicollinearity among the variables in the study and covariate selection, a variance inflation factor (VIF) was used for variable selection. A general rule of thumb is that a VIF value >5 indicates problematic covariance (46). The VIF values for gender, age, race, education level, marital status, family income, energy, BMI, alcohol use, smoking, PA, diabetes, HEI, CKD, and hypertension were all calculated to be less than 5, and therefore these variables were included in Model 3 for adjustment. In addition, we used directed acyclic graphs (www.dagitty.net/dags.html) to show the hypothesized relationships between variables (**Figure 2**). In this study, we observed that the sKlotho data were unevenly distributed and skewed. Considering that the logarithmic transformation helps to stabilize the variance and reduce the effect of outliers when dealing with skewed data, we used the natural logarithm (Ln)sKlotho to make it more suitable for our statistical analysis. This transformation may change the relationship between the variables and requires careful interpretation of the results. Therefore, when reporting the results, we also provide the results of the analyses with untransformed variables so that the reader can compare and assess the impact of the transformation. In the sensitivity analyses, a linear trend test was conducted using the WWI quartile set as the dependent variable to assess its robustness (47). Subgroup analyses were conducted using stratified multivariate regression models with stratification factors including gender, age, education, marital status, family income, HEI, PA, hypertension, diabetes, CKD, alcohol use, smoking, and SII quartile group. Smoothed curve fitting was used in this study to determine if there was a linear relationship between WWI and sKlotho. Finally, this study used Bootstrap to validate the analysis of the mediating role of SII between WWI and sKlotho (48). To maintain data and computational accuracy, this paper ensured that the samples used in the analysis were complete and accurate on all covariates by excluding missing values. All analyses were performed using EmpowerStats (http://www.empowerstats.com), SPSS27.0, and amos24.0. Statistical significance was assessed at a two-sided value of P<0.05. In subgroup analyses, to avoid increasing type I error due to multiple comparisons, false discovery rate control was used in this paper to adjust for multiple testing, and FDR-corrected p<0.1 was considered significant (49).

## 3 Results

### 3.1 Baseline characteristics of participants

A total of 9,506 participants who met the predefined inclusion and exclusion criteria were enrolled in this study, all over 45 years of age. **Table 1** shows the baseline weighted characteristics of the included population stratified by sKlotho quartiles. Within the sKlotho quartiles, there were significant differences in age, gender, race, smoking, alcohol use, SII, HEI, hypertension, diabetes, CKD and education. However, no significant differences were observed in family income, BMI, PA, marital status, and energy.

**Table 1:** Characteristics of included population in the NHANES based on sKlotho tertiles (N = 9,506).

Characteristics	sKlotho Tertiles				*P*-Value
	Q1	Q2	Q3	Q4	
Age(years)	61.29 ± 9.64	60.39 ± 9.43	59.85 ± 9.35	59.36 ± 9.11	<0.001
SII	575.67 ± 357.69	543.45 ± 649.68	532.71 ± 366.35	489.00 ± 294.49	<0.001
WWI	11.38 ± 0.74	11.33 ± 0.72	11.30 ± 0.74	11.25 ± 0.76	<0.001
BMI (kg/m^2^)	30.01 ± 6.33	29.70 ± 6.47	29.64 ± 6.46	29.78 ± 6.78	0.062
WC	103.79 ± 14.98	102.76 ± 15.31	102.07 ± 15.12	101.65 ± 15.72	<0.001
Energy(kcal)	1984.29 ± 883.83	2016.83 ± 904.73	1996.85 ± 909.22	1978.56 ± 897.53	0.616
HEI	51.60 ± 13.38	52.40 ± 13.77	53.07 ± 13.90	52.91 ± 14.36	<0.001
Gender (%)					<0.001
Male	1266 (53.26%)	1246 (52.46%)	1140 (48.02%)	1064 (44.71%)	
Female	1111 (46.74%)	1129 (47.54%)	1234 (51.98%)	1316 (55.29%)	
Race/Ethnicity (%)					<0.001
Mexican American	355 (14.93%)	360 (15.16%)	356 (15.00%)	340 (14.29%)	
Other Hispanic	222 (9.34%)	244 (10.27%)	265 (11.16%)	305 (12.82%)	
Non-Hispanic White	1151 (48.42%)	1186 (49.94%)	1134 (47.77%)	970 (40.76%)	
Non-Hispanic Black	479 (20.15%)	402 (16.93%)	403 (16.98%)	593 (24.92%)	
Other Race	170 (7.15%)	183 (7.71%)	216 (9.10%)	172 (7.23%)	
Education level (%)					0.049
Less than high school	309 (13.00%)	302 (12.72%)	284 (11.96%)	291 (12.23%)	
High school	921 (38.75%)	833 (35.07%)	875 (36.86%)	836 (35.13%)	
More than high school	1147 (48.25%)	1240 (52.21%)	1215 (51.18%)	1253 (52.65%)	
Marital status (%)					0.078
Married/Living with partner	1529 (64.32%)	1504 (63.33%)	1530 (64.45%)	1512 (63.53%)	
Widowed/Divorced/Separated	683 (28.73%)	684 (28.80%)	694 (29.23%)	662 (27.82%)	
Never married	165 (6.94%)	187 (7.87%)	150 (6.32%)	206 (8.66%)	
Family income (%)					0.249
0–1.5	838 (35.25%)	802 (33.77%)	778 (32.77%)	811 (34.08%)	
1.5–3.5	762 (32.06%)	723 (30.44%)	742 (31.26%)	740 (31.09%)	
>3.5	777 (32.69%)	850 (35.79%)	854 (35.97%)	829 (34.83%)	
Diabetes					<0.001
Yes	602 (25.33%)	519 (21.85%)	537 (22.62%)	625 (26.26%)	
No	1775 (74.67%)	1856 (78.15%)	1837 (77.38%)	1755 (73.74%)	
Hypertension (%)					<0.001
Yes	1711 (71.98%)	1583 (66.65%)	1558 (65.63%)	1601 (67.27%)	
No	666 (28.02%)	792 (33.35%)	816 (34.37%)	779 (32.73%	
CKD(%)					<0.001
Yes	419 (17.63%)	260 (10.95%)	199 (8.38%)	168 (7.06%)	
No	1958 (82.37%)	2115 (89.05%)	2175 (91.62%)	2212 (92.94%)	
PA(%)					0.022
[0–600)	886 (37.27%)	817 (34.40%)	795 (33.49%)	868 (36.47%)	
≥600	1491 (62.73%)	1558 (65.60%)	1579 (66.51%)	1512 (63.53%)	
HEI status (%)					0.001
Insufficient	1119 (47.08%)	1081 (45.52%)	1002 (42.21%)	1023 (42.98%)	
Average	1031 (43.37%)	1025 (43.16%)	1095 (46.12%)	1053 (44.24%)	
Optimal	227 (9.55%)	269 (11.33%)	277 (11.67%)	304 (12.77%)	
Smoking (%)					<0.001
Yes	1339 (56.33%)	1245 (52.42%)	1171 (49.33%)	1083 (45.50%)	
No	1038 (43.67%)	1130 (47.58%)	1203 (50.67%)	1297 (54.50%)	
Alcohol use (%)					<0.001
Yes	1796 (75.56%)	1714 (72.17%)	1688 (71.10%)	1565 (65.76%)	
No	581 (24.44%)	661 (27.83%)	686 (28.90%)	815 (34.24%)	

Percentage and mean standard deviation were weighted. Family income(%), the ratio of family income to poverty; SII, systemic immune-inflammation index; WC, waist circumference; BMI, body mass index; HEI, Healthy Eating Index; CKD, chronic kidney disease; PA, physical actibity; WWI, weight-adjusted-waist index.

### 3.2 The associations of SII and WWI with sKlotho

We first performed a weighted multivariate linear regression analysis of the relationship between WWI and Ln-sKlotho, and **Table 2** shows that in the unadjusted model [β (95%CI) = -0.03 (-0.04, -0.02)], the minimally-adjusted model [-0.03 (-0.04, -0.02)] and the fully-adjusted model [-0.03 (-0.04, -0.01)], WWI was negatively correlated with Ln-sKlotho. Sensitivity analysis using WWI as a categorical variable (quartiles) showed that it was still statistically significant (β=-0.04, 95%CI: -0.06- -0.01, *p*=0.0016). Next, we analyzed the relationship between SII and Ln-sKlotho and **Table 2** shows that SII was negatively correlated with Ln-sKlotho in unadjusted, minimally adjusted and fully adjusted models. We also analyzed the relationship between WWI, SII and sKlotho, and **Table 3** shows that both WWI and SII were negatively correlated with sKlotho.

**Table 2:** Association between WWI and Ln-sKlotho.

	Crude Model (Model 1)	Partially Adjusted Model (Model 2)	Fully Adjusted Model (Model 3)
	β (95% CI) *p*-Value	β (95% CI) *p*-Value	β (95% CI) *p*-Value
WWI	-0.03 (-0.04, -0.02) <0.0001	-0.03 (-0.04, -0.02) <0.0001	-0.03 (-0.04, -0.01) <0.0001
WWI quartiles			
Q1	0	0	0
Q2	-0.02 (-0.04, -0.00) 0.0467	-0.01 (-0.03, 0.01) 0.1720	-0.01 (-0.03, 0.01) 0.3255
Q3	-0.04 (-0.06, -0.02) <0.0001	-0.03 (-0.05, -0.01) 0.0012	-0.03 (-0.05, -0.01) 0.0068
Q4	-0.05 (-0.07, -0.03) <0.0001	-0.04 (-0.06, -0.02) <0.0001	-0.04 (-0.06, -0.01) 0.0016
SII	-0.00 (-0.00, -0.00) <0.0001	-0.00 (-0.00, -0.00) <0.0001	-0.00 (-0.00, -0.00) <0.0001
SII quartiles			
Q1	0	0	0
Q2	-0.04 (-0.06, -0.02) 0.0001	-0.03 (-0.05, -0.01) 0.0005	-0.03 (-0.05, -0.01) 0.0007
Q3	-0.05 (-0.07, -0.03) <0.0001	-0.05 (-0.07, -0.03) <0.0001	-0.05 (-0.07, -0.03) <0.0001
Q4	-0.09 (-0.11, -0.08) <0.0001	-0.09 (-0.11, -0.07) <0.0001	-0.08 (-0.10, -0.06) <0.0001

Insensitivity analysis, WWI and SII was converted from a continuous variable to a categorical variable (quartiles).

β, Partial regression coefficient; Cl,confidence interval;SII,systemic immune-inflammation index;WWI,weight-adjusted-waist index.

Model 1, no covariates were adjusted.

Model 2, age, gender, race were adjusted.

Model 3, age, gender, race, marital status, family income, education level, BMI, energy, HEI, PA, alcohol use, smoke, hypertension, diabetes, and CKD were adjusted.

**Table 3:** Association between WWI and sKlotho.

	Crude Model (Model 1)	Partially Adjusted Model (Model 2)	Fully Adjusted Model (Model 3)
	β (95% CI) *p*-Value	β (95% CI) *p*-Value	β (95% CI) *p*-Value
WWI	-24.58 (-32.89, -16.28) <0.0001	-22.97 (-32.01, -13.94) <0.0001	-23.55 (-34.31, -12.79) <0.0001
WWI quartiles			
Q1	0	0	0
Q2	-19.69 (-37.03, -2.35) 0.0260	-13.72 (-31.14, 3.70) 0.1228	-11.42 (-29.12, 6.28) 0.2060
Q3	-37.45 (-54.79, -20.11) <0.0001	-29.84 (-47.66, -12.02) 0.0010	-28.13 (-46.95, -9.31) 0.0034
Q4	-43.29 (-60.63, -25.96) <0.0001	-38.98 (-57.55, -20.40) <0.0001	-36.39 (-57.74, -15.03) 0.0008
SII	-0.05 (-0.06, -0.04) <0.0001	-0.04 (-0.06, -0.03) <0.0001	-0.04 (-0.05, -0.02) <0.0001
SII quartiles			
Q1	0	0	0
Q2	-40.59 (-57.87, -23.31) <0.0001	-35.40 (-52.69, -18.11) <0.0001	-34.24 (-51.40, -17.09) <0.0001
Q3	-55.71 (-72.98, -38.43) <0.0001	-49.86 (-67.22, -32.50) <0.0001	-47.32 (-64.55, -30.08) <0.0001
Q4	-84.22 (-101.50, -66.95) <0.0001	-75.11 (-92.58, -57.64) <0.0001	-69.32 (-86.76, -51.88) <0.0001

Insensitivity analysis, WWI and SII was converted from a continuous variable to a categorical variable (quartiles).

Abbreviations:β, Partial regression coefficient; Cl,confidence interval;SII,systemic immune-inflammation index;WWI,weight-adjusted-waist index.

Model 1, no covariates were adjusted.

Model 2, age, gender, race were adjusted.

Model 3, age, gender, race, marital status, family income, education level, BMI, energy, HEI, PA, alcohol use, smoke, hypertension, diabetes, and CKD were adjusted.

**Figure 3:**
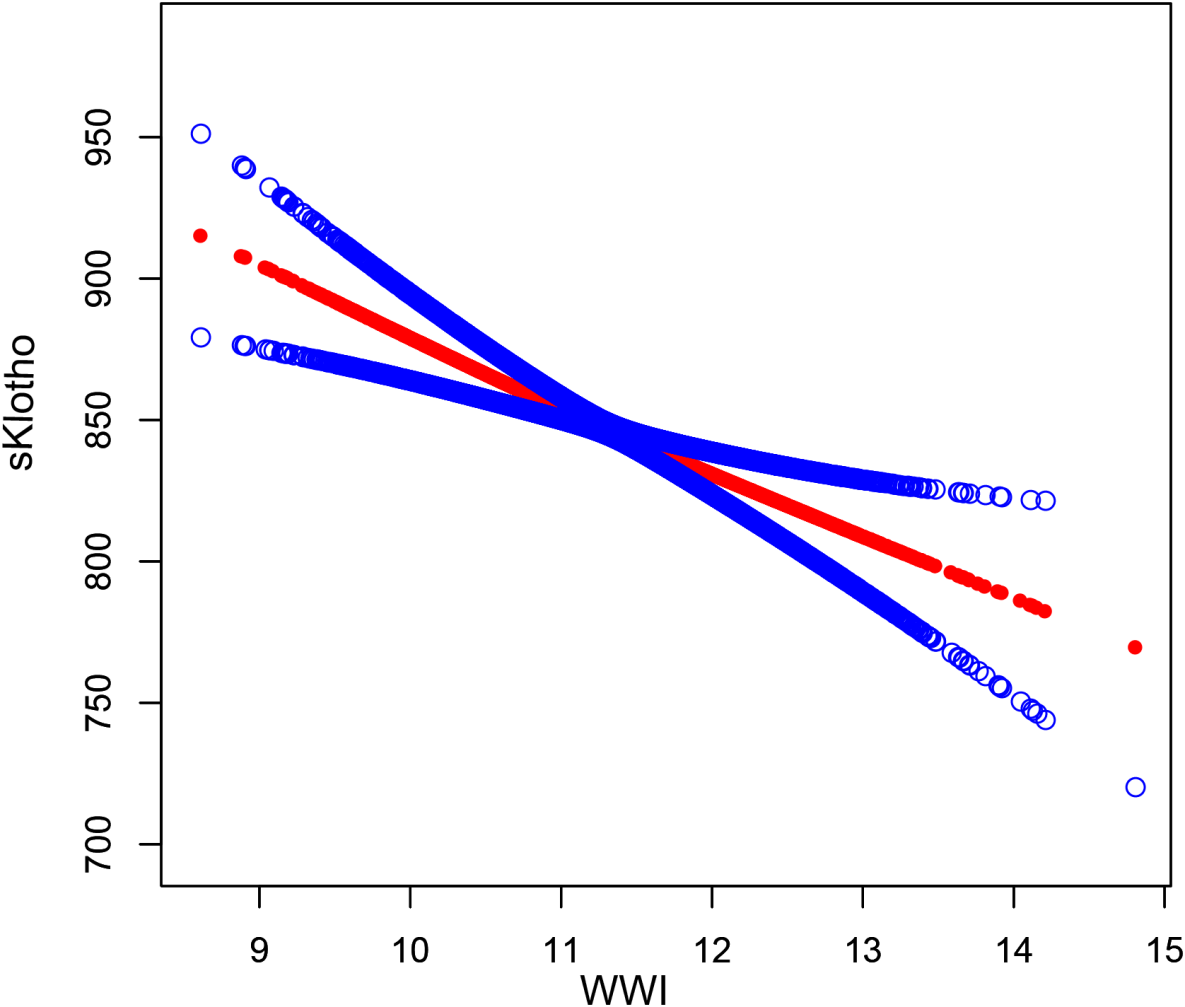
The association between WWl and sKlotho. The solid red line represents the smooth curve fit between variables. Blue bands represent the 95% confidence interval from the fit.

Based on this, subgroup analysis of the relationship between WWI and sKlotho (**Table 4**), gender, age, education, marital status, family income, HEI, PA, hypertension, diabetes, CKD, alcohol use, smoking, and SII did not affect the negative correlation between WWI and sKlotho (FDR-corrected *p*>0.1). Smooth curve fitting showed a negative correlation between WII and sKlotho (**Figure 3**).

**Table 4:** Subgroup analysis of the effect of WWI on sKlotho.

Subgroups	β (95% CI), P-value	FDR-corrected p for interaction
Gender		0.1345
Male	7.57 (-25.43, 10.29) 0.4062	
Female	-35.42 (-48.94, -21.89) <0.0001	
Age (years)		0.7382
[45–64)	-22.41 (-34.66, -10.17) 0.0003	
≥65	-27.65 (-43.45, -11.86) 0.0006	
Education level		0.3136
Less than high school	4.06 (-25.60, 33.72) 0.7883	
High school	-22.03 (-39.89, -4.17) 0.0157	
More than high school	-32.36 (-47.56, -17.16) <0.0001	
Marital status		0.8946
Married/Living with partner	-22.80 (-36.50, -9.10) 0.0011	
Widowed/Divorced/Separated	-23.38 (-42.19, -4.57) 0.0149	
Never married	-34.61 (-70.14, 0.91) 0.0562	
Smoking		0.1746
Yes	-12.42 (-27.28, 2.44) 0.1013	
No	-32.68 (-46.85, -18.52) <0.0001	
Alcohol use		0.1345
Yes	-16.47 (-28.69, -4.25) 0.0083	
No	-37.49 (-53.64, -21.34) <0.0001	
Hypertension		0.7382
Yes	-21.04 (-33.68, -8.41) 0.0011	
No	-27.95 (-45.69, -10.22) 0.0020	
CKD (%)		0.5666
Yes	-8.44 (-37.50, 20.61) 0.5689	
No	-25.43 (-36.73, -14.13) <0.0001	
PA (%)		0.8406
0–600	-21.01 (-36.98, -5.04) 0.0099	
>600	-24.51 (-37.22, -11.80) 0.0002	
HEI status (%)		0.7382
Insufficient	-24.81 (-40.63, -8.99) 0.0021	
Average	-19.06 (-33.53, -4.60) 0.0098	
Optimal	-57.73 (-123.08, 7.61) 0.0833	
Diabetes		0.5517
Yes	-33.21 (-52.85, -13.56) 0.0009	
No	-19.58 (-31.46, -7.70) 0.0012	
Family income		0.5666
(0–1.5)	-30.42 (-47.42, -13.42) 0.0005	
[1.5–3.5)	-13.60 (-31.27, 4.08) 0.1316	
≥3.5	-29.11 (-46.66, -11.56) 0.0012	
SII quartiles		0.9827
Q1	-20.89 (-42.41, 0.63) 0.0572	
Q2	-20.74 (-41.52, 0.04) 0.0505	
Q3	-25.59 (-46.12, -5.06) 0.0146	
Q4	-20.60 (-40.64, -0.56) 0.0440	

β, Partial regression coefficient; Cl, confidence interval; WWI, weight-adjusted-waist index.

### 3.3 Analysis of the mediating effect of SII on the association between WWI and sKlotho

**Figure 4:**
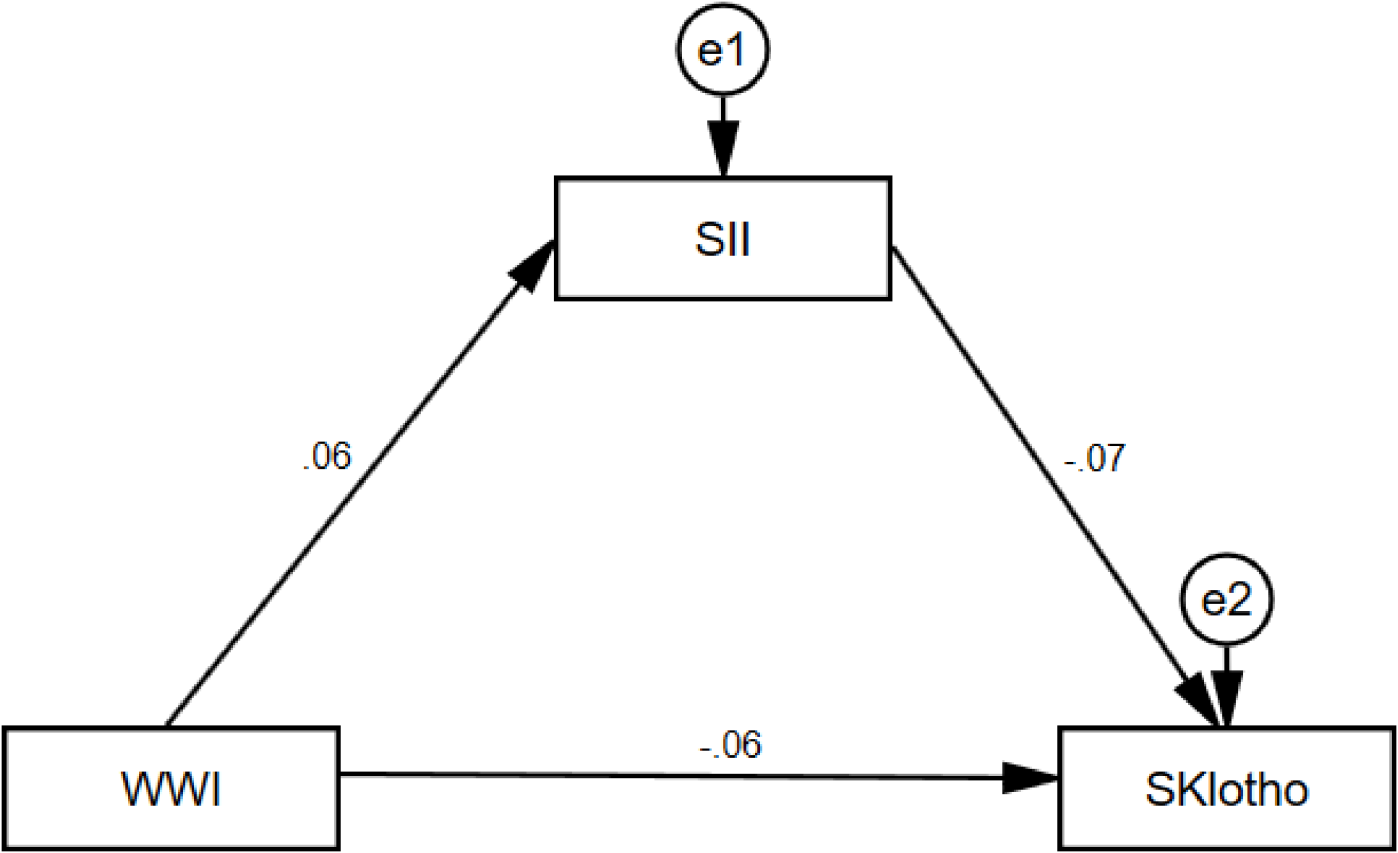
WWI, weight-adjusted-waist index; SII, systemic immune-inflammation index. Total effect -0.059; Direct effect -0.055; Indirect effect -0.004.

To explore the intrinsic mechanism of the significant negative effect of WWI on sKlotho, SII was further introduced as a mediating variable to substitute into the structural equation modeling in the study. The results are shown in **Figure 4**, in which it can be seen that WWI has a significant positive predictive effect on SII (β=0.06, t=2.58, *p*<0.01), and the 95% confidence intervals (hereafter collectively referred to as the “95% confidence intervals”) of the bias-corrected nonparametric percentile-based bootstrap method are [0.021, 0.105], SII had a significant negative predictive effect on sKlotho (β=-0.07, t=-4.79, *p*<0.001) with a 95% confidence interval of [-0.095, -0.046], while WWI had a significant negative predictive effect on sKlotho (β=-0.06, t=-5.50, *p*<0.001) with a 95% confidence interval of [-0.077, -0.035]. The mediating effect of SII in the effect of WWI on sKlotho was -0.004, with a 95% confidence interval of [-0.009, -0.001] excluding 0, indicating that the mediating effect of SII in the effect of WWI on sKlotho was significant. In summary, it can be concluded that the mediation model is partial. The specific results are shown in **Table 5**.

**Table 5:** Using the bootstrap method to test the mediating effect.

	Effect value	S.E.	LLCI	ULCL	Effect size
Total effect	-0.059	0.010	-0.080	-0.039	
Direct effect	-0.055	0.010	-0.077	-0.035	93.32%
Indirect effect	-0.004	0.003	-0.009	-0.001	6.78%

## 4 Discussion

This study investigated the relationship between WWI and SII and sKlotho in the United States over the age of 45. To our knowledge, this is the first study to assess the relationship between WWI and SII and sKlotho. We found that WWI was negatively associated with sKlotho in both crude and adjusted models. Sensitivity analyses with WWI as quartile also showed a correlation between WWI (β=-0.04, 95% CI: -0.06- -0.01, *p*=0.0016) and sKlotho. Subgroup analyses showed that gender, age, education, marital status, income, hypertension, diabetes, alcohol consumption, smoking status, and SII did not affect the negative correlation between WWI and sKlotho (FDR-corrected *p*>0.1) and that SII mediated the relationship between WWI and sKlotho. In future studies, we also recommend that other interested researchers measure sKlotho in patients who have lost weight through dieting or metabolic bariatric surgery to better analyze the relationship between obesity and aging.

The results of this study are similar to other studies, Jian Cui et al. showed a negative correlation between visceral obesity as measured by VAI and the anti-aging protein klotho (50). Taylor Landry et al. showed an inverse correlation between cerebrospinal fluid α-klotho and BMI (51). Multifactorial linear regression of Ping Chen et al. showed a multifactorial adjusted The multifactorial linear regression of Ping Chen et al. showed that high sKlotho levels were associated with low SII levels after multifactorial adjustment (β=-0.08,95% CI: -0.10- -0.05, *p*< 0.01) (52). Zhu Wen et al. also showed that SII levels were negatively correlated with serum klotho protein concentration in U.S. adults (53).

However, the mechanism behind the link between WWI and sKlotho remains unclear. Previous systematic assessments have shown that obesity may contribute to inflammation in several ways, on the one hand, adipose tissue is not only a storage of energy in the body, but is also capable of secreting a variety of adipokines (known as adipokines), including leptin, lipocalin, tumor necrosis factor-alpha (TNF-alpha), and interleukin-6 (IL-6), among others. In the state of obesity, the secretion of these adipokines is imbalanced, the expression of pro-inflammatory factors increases and the expression of anti-inflammatory factors decreases, leading to a state of chronic low-grade inflammation (54, 55). On the other hand, obese individuals often have insulin resistance, which can lead to elevated blood glucose levels, thus inducing inflammatory responses. Insulin resistance reduces the anti-inflammatory effect of insulin, leading to chronic inflammation (56). In addition, Ma TC et al. found a negative correlation between DII (Dietary Inflammatory Index) and sKlotho plasma levels and hypothesized that chronic inflammation induced by a pro-inflammatory dietary pattern could reduce plasma sKlotho level and that chronic inflammation induced by a pro-inflammatory dietary pattern could reduce serum klotho levels (57). In addition, Ma TC et al. found a negative correlation between DII (Dietary Inflammatory Index) and sKlotho plasma levels and hypothesized that chronic inflammation induced by a pro-inflammatory dietary pattern could reduce plasma sKlotho level and that chronic inflammation induced by a pro-inflammatory dietary pattern could reduce serum klotho levels (58). However, the underlying biological mechanisms between SII and sKlotho are not fully understood, and Moreno JA et al. showed that inflammatory cytokines (TWEAK and TNFα) downregulate Klotho expression by inflammatory cytokines including TWEAK and TNF through a transcription factor nuclear factor kappaB (NFκB)-dependent mechanism (59). Fitzpatrick EA et al. showed that downregulation of renal Klotho expression was associated with the amplification of inflammation in the kidney of diabetic mice (60) Whereas Klotho proteins have anti-inflammatory and anti-oxidative stress effects, a decrease in Klotho may lead to an increase in intracellular oxidative stress, which in turn promotes cellular senescence and tissue dysfunction. In our study, we found that the 6.78% relationship between WWI and sKlotho appeared to be mediated by SII. Although the mediating effect is numerically small, previous studies have shown that even if the mediating effect is small, it may still be significant. Sun Fei et al. found that SII mediated 5.08% of the correlation between WWI and depression (61). Jing Lin et al. found that 2.7% of the correlation between depression and premature coronary artery disease was mediated by SII (62). Therefore, it can be seen that SII has a certain mediating effect in the correlation of many diseases. These results suggest that SII may play a key mediating role in the relationship between WWI and sKlotho and that decreasing the inflammatory status of obese patients may increase sKlotho levels, which in turn delays the likelihood of obesity-induced aging. Of course this needs to be further verified in the clinic.

The strength of our study is that it is based on national data and the results are broadly applicable to the general population in the United States. Regression analyses adjusted for covariates, and the large sample size allowed us to perform sensitivity and subgroup analyses to confirm the robustness of the results. Finally, the mediating role of SII between WWI and sKlotho was validated. However, there are still some limitations that need to be declared. This is a cross-sectional study, which can only explore the relationship between WWI, SII and sKlotho to provide clues for etiologic studies, and cannot validate the causal relationship between disease and etiology. Based on the results of this study, we suggest that future longitudinal studies could focus on the long-term effects of WWI and SII on sKlotho levels, especially the differences in different age and sex groups. At the same time, Mendelian randomization analysis should be combined to verify whether these associations are causal in nature. This will not only help to reveal the roles of WWI and SII in aging-related diseases, but may also provide a theoretical basis for the development of new intervention strategies. Also although we have adjusted for several potential covariates, many factors still influence sKlotho, and we cannot completely rule out the influence of other potential confounders, such as other social, environmental, and weight loss drug use variables. Our study was based on U.S. adults age 45 and older, so it remains to be seen whether these results apply to young adults and other racial groups.

## 5 Conclusion

This study demonstrated an association between WWI, SII and sKlotho, and that SII plays an important role in mediating the relationship between WWI and sKlotho levels. These findings inform the improvement of sKlotho levels and the prevention of aging from the perspectives of obesity indicators and systemic inflammation indices, and clinical healthcare professionals should pay more attention to obesity and systemic inflammation levels in middle-aged and elderly populations to help them better prevent aging. However, due to the limitations of the study design, we were unable to determine the causal relationship between these variables. Further validation through longitudinal studies and Mendelian randomization analyses is needed in the future.

## Data Availability

Publicly available datasets were analyzed in this study. This data can be found here: www.cdc.gov/nchs/NHANEs/.
